# Clinical Remarks upon Surgical Cases in the Buffalo General Hospital—Necrosis of the Tibia, with Contraction of Tendo Achillis—Scirrhus Tumor

**Published:** 1864-12

**Authors:** J. F. Miner


ART. V.—*Clinical Remarks upon Surgical Cases in the Buffalo General Hospital—Necrosis of the Tibia, with contraction of Tendo Achillis—Scirrhus Tumor.* By J. F. Miner, M. D.

November 9, 1864.

The first patient which I have to present for operation is a young lad 12 years of age, of delicate constitution, who has been sent to me by Drs. Allen and Havens of Aurora, who entertain as I suppose by this circumstance, the opinion that something can be done by operation for the removal of the dead bone which you observe protruding from the tibia. I most fully agree with them in this opinion, and propose opening down upon the tibia its whole, or nearly its whole length, with the view of extracting what is called a sequestrum from the interior of the bone. You will observe that this dead bone, or sequestrum, is the old or original tibia, that the processes both of the death of bone and repair, have been going on at the same time, and that while the tibia has become dead, another bone has grown around it. These openings, which we call cloaca, afford exit to the purulent collections, and yet are not sufficient to admit the extraction and removal of the dead bone, which is imprisoned within thick, dense, unyielding, bony walls. That this process of repair should go on, springing either from healthy bone above or below, as some suppose, or from the separated periosteum, as is believed by others, is truly remarkable—that it should go on more rapidly and more perfectly with dead bone still present, keeping up inflammatory and ulcerative action, than it is ever observed to do when the old bone is removed entire, is a fact so suggestive that it should not be inattentively considered, though possibly the explanation is not difficult. It appears probable to me that the periosteum has much to do in this matter of the re-production of bone. I have seen bone re-produced when removed by operation, in cases only where the periosteum was left entire. In these cases of necrosis the dead bone separates from its periosteal covering, and leaves it nearly uninjured, the bone yet remaining prevents deformity, and forms a base upon which, or around which, nature builds a new and living structure. This new bone is unlike the old—is firmer and more dense—in time becomes almost as firm and solid as ivory. These openings, or cloaca, which communicate with the dead bone beneath, I propose to enlarge, or rather I will cut away the new bone sufficient to allow removal of the necrosed portion, which extends the whole length of the tibia. We shall then have only living structures over which nature may possibly replace a healthy skin, and our little patient yet enjoy the luxury of being perfectly sound, with a new tibia greatly enlarged, but equally useful as the old. You may ask how it is that these round, smooth openings are left in the newly formed bone? Why is it not one solid entire bony case? I think that the ulcerative action has destroyed the periosteum over these places which in part accounts for this condition. Again there is a constant discharge, which might and does no doubt keep open these outlets; nature in such cases always provides for herself. This condition of disease does not extend into the knee above, or into the ankle-joint below, and though it looks to-day as though amputation was the only remedy, still that is a dreadful necessity, and we should hardly be justified in such a measure, until we had given this more conservative and more difficult operation a fair trial. Now I have not only removed a great amount of dead bone from the interior of the tibia, but I propose also to divide the Tendo Achillis, which from the irritation of the disease, has become so shortened as to draw the heel up, and cause the patient to rest only the toes upon the floor. This has been so for years, and is caused by reflex nervous influence upon the muscular tissue above. Possibly extension might partially remedy it, but our little patient is in no good condition to admit of extension, and as the tendon will unite again, we deem it safer, and on the whole more sure to effect relief from deformity, to divide the tendon and immediately place the foot in natural position. This deformity is called sometimes *talipes equinus*—horse foot, and is a condition not often hereditary—existing at birth, but acquired, as in the present instance. The operation for its relief is simple and safe—is not likely to be attended by any unfavorable results. After division of the tendon, the toes are easily elevated and the heel brought down; it is retained in place by a band fastened to the thigh above the knee. The posterior tibial artery passes in the near neighborhood of this tendon, and fears are often entertained of wounding it in this operation; it seems to me there is little or no danger of this. I have already told you that this tendon when divided will unite again; it will become connected with a firm and dense structure, though I hardly think that its mobility is as great after its division and attachment; the cicatrix is apt to involve other parts and form attachments, and the cord is not smooth and perfect. Various methods of dividing tendons, is such cases have been proposed with the view of furthering the re-union; the ones of chief value, which are to be mentioned, are partial division and extension, and very oblique or longitudinal section, the tendon to be slid upon itself. I think that division, however effected, will usually be attended by very similar results in the end, and that the operation which is the most simple, and makes the least wound, is the best.

I repeat a brief description of what you have seen—describe in few words, a long and tedious operation, in removal of the dead bone, and in division of the tendon, a safe and simple one. I have then operated for necrosis of the tibia and removed the sequestrium by cutting the various cloaca into one continuous elongated opening, through which all dead bone has been removed; and also I have divided the Tendo Achillis for the purpose of allowing the foot to be placed in natural position—position of greater comfort and usefulness. This is a case of great interest, and if it should progress favorably will illustrate in remarkable manner how much nature is to be depended upon in restoration from disease, and how we can only act wisely and safely, while as physicians and surgeons we follow her guidance.*

* The case progressed rapidly and favorably; healthy granulations soon covered the extensive surface of cut bone; the integument united mostly, and November 28th, about three weeks after the operation, the boy returned to his home in the country, with every prospect of a perfectly satisfactory result.

*Scirrhus Tumor.*—This is an operation for re-removal of malignant disease of the breast. The first operation was before you last year, and on that account it is the more interesting; it shows that though we extirpate scirrhus growths, entire, even at early period, still they are liable to return. The propriety of operative interference is on this account questioned by some surgeons. If it is done early, before the system suffers perceptibly, or the neighboring glands enlarge, it appears probable that nothing is lost; in some cases it seems certain, almost, that much is gained. It has re-appeared as is more common, in the cicatrix, made by removal of the breast, and we remove it again, and again and again, as I propose, should it return, fighting this relentless and perhaps unconquerable foe, until our "supplies" are cut off, or our patient "taken prisoner."

